# Supplementary material for: Integrase anchors viral RNA to the HIV-1 capsid interior
Source: Nature. 2026 Feb 18;652(8111):1068–75. doi: 10.1038/s41586-026-10154-x (PMC13102720; doi:10.1038/s41586-026-10154-x)

nature portfolio

https://doi.org/10.1038/s41586-026-10154-x

Supplementary information

Integrase anchors viral RNA to the HIV-1 capsid interior

In the format provided by the authors and unedited

Uncropped gels from Extended Fig. 2a.

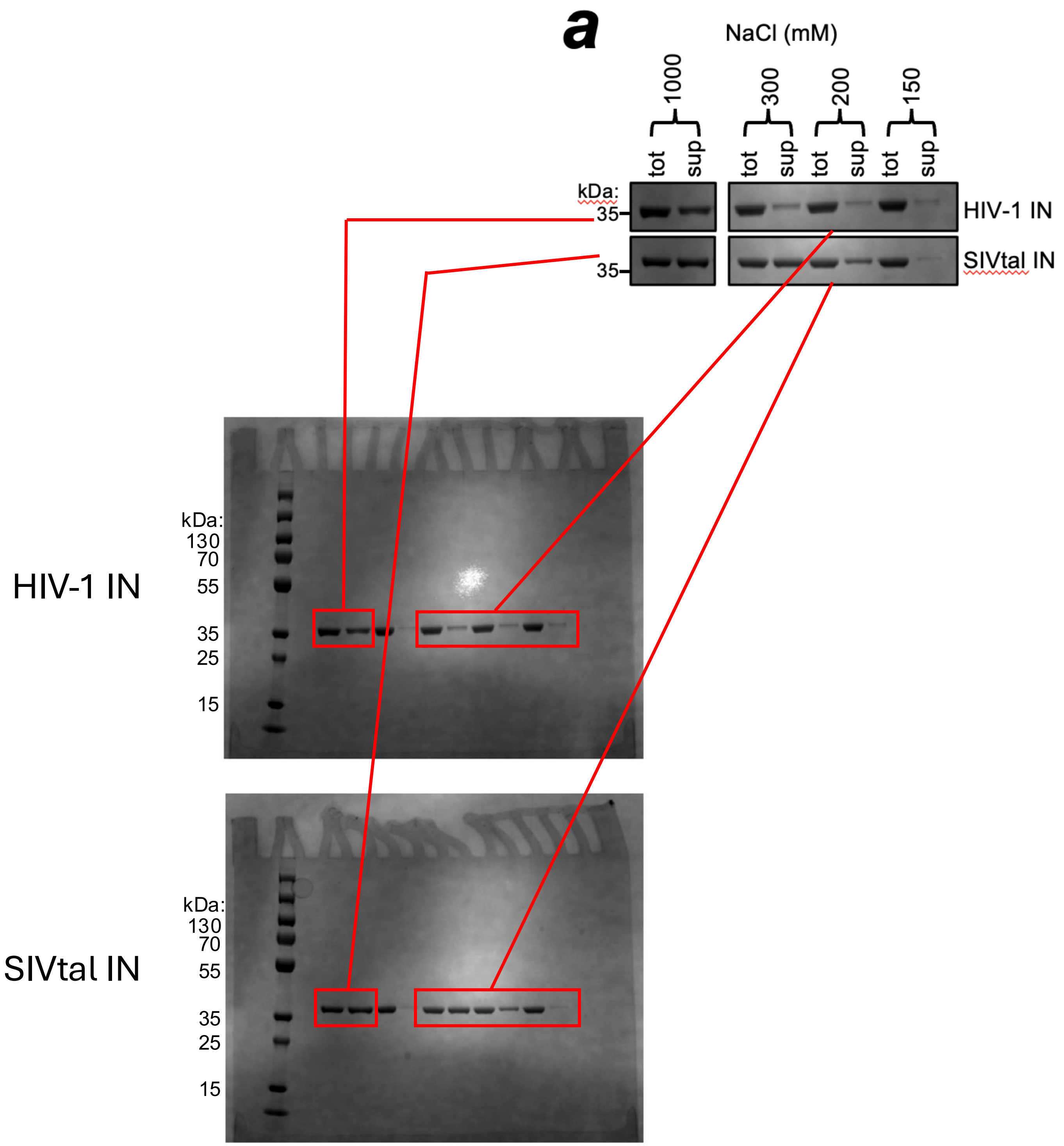

Uncropped blots and color image from Extended Fig. 4c.

Anti-IN, polyclonal:

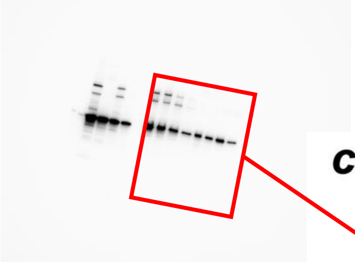

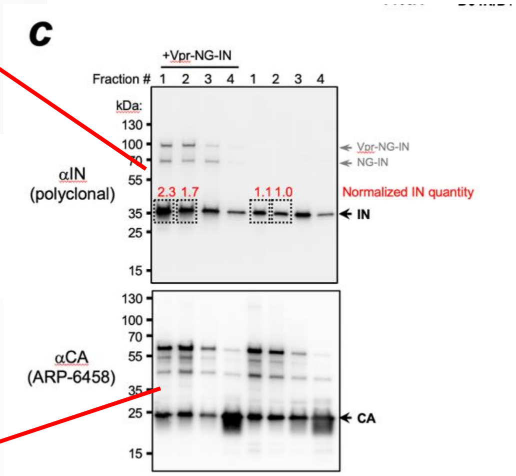

Anti-CA, ARP-6458:

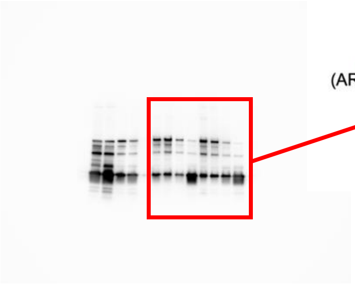

ImageJ quantification of core fractions:

	Area	
Lane 1	14894.016	
Lane 2	10828.995	
Lane 5	7531.146	
Lane 6	5983.539	

ImageJ quantification of core fractions:

	Area	
Lane 1	11595.016	
Lane 2	10860.359	
Lane 5	12080.309	
Lane 6	10494.187	

Quantification using ImageJ:				
	IN sinal	CA signal	ratio (IN/CA)	relative ratio
lane1	14894	11595	1.28	2.25
lane2	10829	10860	1.00	1.75
lane5	7531	12080	0.62	1.09
lane6	5984	10494	0.57	1.00

NeonGreen fluorescence:

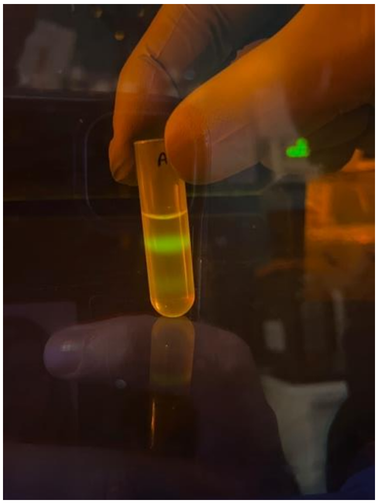

Uncropped blots from Extended Fig. 7d.

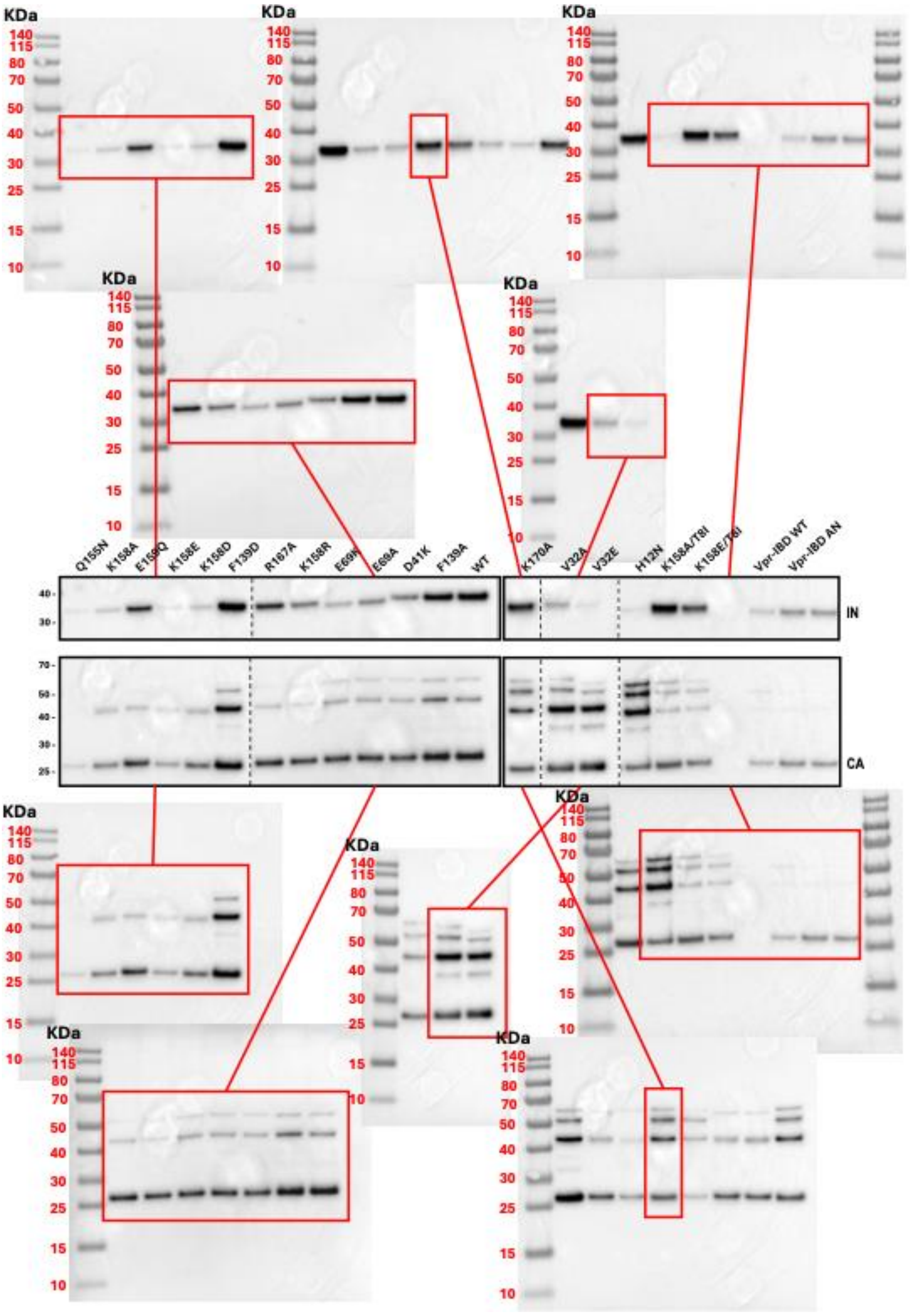

Supplement: Supplementary file 1 — Uncropped gel and blot images. [file 41586_2026_10154_MOESM1_ESM.pdf]
