# Supplementary material for: Integrase anchors viral RNA to the HIV-1 capsid interior
Source: Nature. 2026 Feb 18;652(8111):1068–75. doi: 10.1038/s41586-026-10154-x (PMC13102720; doi:10.1038/s41586-026-10154-x)
Supplement: Supplementary file 2 — Reporting Summary [file 41586_2026_10154_MOESM2_ESM.pdf]

nature portfolio

Corresponding author(s): Peter Cherepanov
Alan Engelman
Juan Perilla

Last updated by author(s): 5 December 2025

# Reporting Summary

Nature Portfolio wishes to improve the reproducibility of the work that we publish. This form provides structure for consistency and transparency in reporting. For further information on Nature Portfolio policies, see our Editorial Policies and the Editorial Policy Checklist.

## Statistics

For all statistical analyses, confirm that the following items are present in the figure legend, table legend, main text, or Methods section.

| n/a | Confirmed | |
|---|---|---|
| ☐ | ☒ | The exact sample size (*n*) for each experimental group/condition, given as a discrete number and unit of measurement |
| ☐ | ☒ | A statement on whether measurements were taken from distinct samples or whether the same sample was measured repeatedly |
| ☐ | ☒ | The statistical test(s) used AND whether they are one- or two-sided<br>*Only common tests should be described solely by name; describe more complex techniques in the Methods section.* |
| ☒ | ☐ | A description of all covariates tested |
| ☐ | ☒ | A description of any assumptions or corrections, such as tests of normality and adjustment for multiple comparisons |
| ☐ | ☒ | A full description of the statistical parameters including central tendency (e.g. means) or other basic estimates (e.g. regression coefficient) AND variation (e.g. standard deviation) or associated estimates of uncertainty (e.g. confidence intervals) |
| ☐ | ☒ | For null hypothesis testing, the test statistic (e.g. *F*, *t*, *r*) with confidence intervals, effect sizes, degrees of freedom and *P* value noted<br>*Give P values as exact values whenever suitable.* |
| ☒ | ☐ | For Bayesian analysis, information on the choice of priors and Markov chain Monte Carlo settings |
| ☒ | ☐ | For hierarchical and complex designs, identification of the appropriate level for tests and full reporting of outcomes |
| ☒ | ☐ | Estimates of effect sizes (e.g. Cohen's *d*, Pearson's *r*), indicating how they were calculated |

*Our web collection on statistics for biologists contains articles on many of the points above.*

## Software and code

Policy information about availability of computer code

| | |
|---|---|
| Data collection | Cryo-EM images were recorded using EPU version 3.10 and Tomography version 5.21, available from Thermo Fisher.<br>Biolayer interferometry data were recorded using Octet BLI Discovery software version 12.2 (Sartorius).<br>NAMD versions 3.0.1 and 3.1 alpha were used for MD simulations, as described. |
| Data analysis | Data analysis and validation was conducted using commercially or publicly available software (all references are given in the manuscript).<br>MSe data were analyzed using Protein Lynx Global Server software (Waters).<br>HDX data were analyzed using DynamX software version 3.0 (Waters)<br>Cryo-EM image processing and 3D volume reconstruction: Relion version 4 and cryoSPARC version 4.6.2.<br>Motion correction was done using Relion-4.0, MotionCor2 version 1.4.0, or WarpTools version 2.0.0 as described.<br>CTF estimation was done using Gctf-v1.18 or WarpTools version 2.0.0 as described.<br>Particles were picked using crYOLO version 1.9.6, Gautomatch version 0.56, and Topaz version 0.2.5a, as described.<br>YOLOv11 (Ultralytics) was used to identify cores in micrographs; Roboflow web application (https://roboflow.com/) was used to build training dataset for YOLOv11.<br>Cryo-ET data processing: WarpTools version 2.0.0 and IMOD version 5.1.1.<br>UCSF Chimera version 1.17.3 was used for rigid body docking of atomistic models into cryo-EM maps and to create images.<br>Namdinator web application (https://namdinator.au.dk/) was used for preliminary refinement of docked structural models.<br>ChimeraX version 1.7 with AritiaX plugin was used to visualize tomograms.<br>PyMOL version 2.4.1 was used to create illustrations with structural models.<br>Tomogram denoising was done using DeepDeWedge software.<br>Particle picking was done using Cryolo version 1.9.9, Gautomatch version 0.56, Topaz 0.2.5a, or Pytom version 0.11.1.<br>Cryo-EM map post-processing: CryoSPARC version 4.6.2 and EMReady version 2. |

Cryo-EM structure real-space refinement: Coot version 0.9.8 and Phenix version 1.21.2.
Final model validation: MolProbity version 4.5 and Phenix version 1.21.2.
MALLS data were analyzed using ASTRA version 7.3.2 software (Wyatt Technology).
MODELLER version 10.6 was used to build missing residues to prepare models for molecular dynamics.
Visual Molecular Dynamics (VMD) version 1.9.4a57, compiled to work with python 3.9.
Scripts used for analysis of the molecular dynamics simulation trajectories can be found at https://doi.org/10.5281/zenodo.15866690.
ImageJ version 1.54g was used for semi-quantitative analysis of CA and IN signals on Western blots.
Virology data statistics was done with GraphPad Prism version 10.6.0
RNA structures were predicted using RNAfold web server (http://rna.tbi.univie.ac.at/cgi-bin/RNAWebSuite/RNAfold.cgi).
Protease cleavage sites were predicted using PROSPERousPlus (http://prosperousplus.unimelb-biotools.cloud.edu.au/) and HIVcleave (http://www.csbio. sjtu.edu.cn/bioinf/HIV/) web servers.

For manuscripts utilizing custom algorithms or software that are central to the research but not yet described in published literature, software must be made available to editors and reviewers. We strongly encourage code deposition in a community repository (e.g. GitHub). See the Nature Portfolio guidelines for submitting code & software for further information.

## Data

Policy information about availability of data

All manuscripts must include a data availability statement. This statement should provide the following information, where applicable:

- Accession codes, unique identifiers, or web links for publicly available datasets
- A description of any restrictions on data availability
- For clinical datasets or third party data, please ensure that the statement adheres to our policy

The cryo-EM and cryo-ET reconstructions were deposited with the EMDB under accession codes EMD-54067, EMD-54068, EMD-54070, EMD-54071, EMD-55409 and refined coordinates with the PDB under accession codes 9RMU and 9RMX (Extended Data Table 1). Raw tilt series data are available in EMPIAR under accession code EMPIAR-13078. HDX-MS data are available via ProteomeXchange with identifier PXD070910. All input parameters, structures and configuration parameters necessary for performing the molecular dynamics simulations presented in this work are freely available in Zenodo (https://doi.org/10.5281/zenodo.15866690). Structures used as starting models or for comparative analyses in this work are freely available from the PDB: 1K6Y, 2B4J, 5TC2, 6ES8, 6SKK, 7ASH, 7Z1Z, 8A1P, 9C9M.

## Research involving human participants, their data, or biological material

Policy information about studies with human participants or human data. See also policy information about sex, gender (identity/presentation), and sexual orientation and race, ethnicity and racism.

| | |
|---|---|
| Reporting on sex and gender | N/A |
| Reporting on race, ethnicity, or other socially relevant groupings | N/A |
| Population characteristics | N/A |
| Recruitment | N/A |
| Ethics oversight | N/A |

Note that full information on the approval of the study protocol must also be provided in the manuscript.

# Field-specific reporting

Please select the one below that is the best fit for your research. If you are not sure, read the appropriate sections before making your selection.

☒ Life sciences ☐ Behavioural & social sciences ☐ Ecological, evolutionary & environmental sciences

For a reference copy of the document with all sections, see nature.com/documents/nr-reporting-summary-flat.pdf

# Life sciences study design

All studies must disclose on these points even when the disclosure is negative.

| | |
|---|---|
| Sample size | Viruses were derived from transfected HEK293T cells in vitro. Final sample sizes were defined by the number of mutants under study. Sample sizes for individual experiments were in large part determined empirically. For example, to determine virus morphology by TEM, individual experiments contained 6 samples to match the capacity of the ultracentrifuge rotor.<br>For the molecular dynamics simulations, n=4 independent 1 microsecond trajectories were utilized for the IN-CA contact analysis. Using n>3 simulation replicas and microsecond length are standard for all-atom molecular dynamics studies. |
| Data exclusions | During cryo-EM image analysis, particle images belonging to 2D or 3D classes representing noise or lacking identifiable features were excluded, as explained in the Methods section. |

| | |
|---|---|
| | No data were excluded from virology experiments or from MD simulations. |
| Replication | Fig. 4d: The experiments were repeated twice with similar results, aggregated results are shown in the figure, and results of separate trials are given in Source Data.<br>Extended Data Fig. 2a: Increased solubility of SIVtal IN relative to HIV-1 IN was consistently observed in multiple (n>3) independent repeats. Concordantly, unlike HIV-1 IN, SIVtal IN can be purified in the absence of CHAPS (see Methods).<br>Extended Data Fig 3a: Assembly of SIVtal IN complexes with TAR and GA18 RNA complexes were repeated 3 times (on different days), with filaments observed each time. Similarly, SIVtal IN in the absence of RNA was imaged 3 independent times, and no filaments were observed by cryo-EM.<br>Extended Data Fig. 4c: Purification of the HIV-1 cores in sucrose gradients is a routine procedure in our laboratories. Comparative immunoblotting of cores and without Vpr-NeonGreen-IN was done twice with similar results. Importantly, the fusion construct does not significantly affect HIV-1 infectivity and moreover rescues infectivity of HIV-1 IN mutants (ED Fig. 4b).<br>Cryo-EM/ET datasets for image processing and structural characterization were acquired from the best sample (one vitrified grid) identified by screening on lower power microscopes. Cryo-EM and cryo-ET reconstructions were refined using pairs of independent half-sets.<br>Final virus release, infection, and reverse transcription datasets were based on minimally three independent experiments, with each experiment containing technical duplicate samples. All attempts of experimental repeats were successful.<br>Some immunoblotting samples were analyzed two independent times, and both repeats were successful. Such samples were omitted from statistical evaluations.<br>MD simulations were collected from four independent replicas. |
| Randomization | Individual experiments were defined by the number of physically acceptable experimental samples. As discussed above, this sample size could be affected by the number of centrifuge buckets. Samples were semi-randomized based on the fact that the identity of precise samples was not always the same between experiments. Different random seeds were utilized to initialize each independent MD simulation replica.<br><br>Randomization was employed during cryo-EM 3D refinement in Relion and CryoSPARC, which use independent random subsets of particle images to avoid over-refinement. |
| Blinding | Due to practical constraints, investigators were not blinded to sample identity and outcome assessment. |

# Reporting for specific materials, systems and methods

We require information from authors about some types of materials, experimental systems and methods used in many studies. Here, indicate whether each material, system or method listed is relevant to your study. If you are not sure if a list item applies to your research, read the appropriate section before selecting a response.

## Materials & experimental systems

| n/a | Involved in the study | |
|---|---|---|
| ☐ | ☒ | Antibodies |
| ☐ | ☒ | Eukaryotic cell lines |
| ☒ | ☐ | Palaeontology and archaeology |
| ☒ | ☐ | Animals and other organisms |
| ☒ | ☐ | Clinical data |
| ☒ | ☐ | Dual use research of concern |
| ☒ | ☐ | Plants |

## Methods

| n/a | Involved in the study | |
|---|---|---|
| ☒ | ☐ | ChIP-seq |
| ☒ | ☐ | Flow cytometry |
| ☒ | ☐ | MRI-based neuroimaging |

## Antibodies

| | |
|---|---|
| Antibodies used | HIV-1 capsid, monoclonal AG3.0, NIH HIV Reagent Program, Division of AIDS, NIAID, cat. no. ARP-4121, lot. no. 70052504<br>HIV-1 capsid, monoclonal ARP-6458, NIH HIV Reagent Program, Division of AIDS, NIAID, cat. no. #24-3, lot. no. 130055<br>HIV-1 integrase, polyclonal antibodies generated in-house<br>Polyclonal Goat Anti-Rabbit Immunoglobulins/HRP, Agilent, cat. no. P0448, lot. no. 41723082<br>Polyclonal Swine Anti-Rabbit Immunoglobilins/HRP, Dako, cat. no. P0399, lot no. 20028547<br>Polyclonal Rabbit Anti-Mouse Immunoglobulins/HRP, Dako, cat. no. P0161, lot. no. 20044780<br>Polyclonal Goat Anti-Mouse Immunoglobulins/HRP, Dako, cat. no. P0447, lot. no. 20058518 |
| Validation | AG3.0 and ARP-6458 are widely used in the field (e.g., PMID: 22153299; 26586435) and revealed the appropriately-sized band via immunoblotting.<br>The HIV-1 integrase antibodies were validated at the time of production via the ability to detect purified recombinant integrase as a single band via immunoblotting and has since been used by others in their research (PMIDs: 33351861 and 10877832).<br><br>Species reactivity and suitability for Western blotting of conjugated secondary antibodies was validated by the commercial suppliers (Agilent/Dako) and by many publications, which can be found on CiteAb resource.<br>P0448: https://www.citeab.com/antibodies/3288347-p0448-goat-anti-rabbit-immunoglobulins-hrp-affinity?des=e8124065ecc857ce<br>P0399: https://www.citeab.com/antibodies/3288354-p0399-swine-anti-rabbit-immunoglobulins-hrp-affinit?des=5d02b793f06e63a5<br>P0161: https://www.citeab.com/antibodies/3288338-p0161-rabbit-anti-mouse-immunoglobulins-hrp-ig-frac?des=d4cc0efd2934bd69<br>P0447: https://www.citeab.com/antibodies/3288336-p0447-goat-anti-mouse-immunoglobulins-hrp-affinity?des=b716b61c45a8fd36 |

## Eukaryotic cell lines

Policy information about cell lines and Sex and Gender in Research

| | |
|---|---|
| Cell line source(s) | HEK293T cells were obtained from ATCC (cat. no. CRL-3216).<br>Lenti-X HEK293T cells were obtained from Takara Bio (cat. no. 632180). |
| Authentication | Cells were not independently authenticated. |
| Mycoplasma contamination | HEK293T cells were negative for mycoplasma as evidenced via regular monthly testing with MycoAlert mycoplasma detection kit (Lonza, cat. no. LT07-218).<br><br>Lenti-X HEK293T cells were negatuve by PCR using PHOENIXDX® MYCOPLASMA MIX (Procomcure Biotech, cat. no. PCCSKU15209). |
| Commonly misidentified lines<br>(See ICLAC register) | No commonly misidentified cell lines were used in this study. |

## Plants

| | |
|---|---|
| Seed stocks | N/A |
| Novel plant genotypes | N/A |
| Authentication | N/A |
